# Proneoplastic effects of PGE_2 _mediated by EP4 receptor in colorectal cancer

**DOI:** 10.1186/1471-2407-9-207

**Published:** 2009-06-26

**Authors:** Glen A Doherty, Sinead M Byrne, Eamonn S Molloy, Vikrum Malhotra, Sandra C Austin, Elaine W Kay, Frank E Murray, Desmond J Fitzgerald

**Affiliations:** 1 Molecular Medicine Group, Conway Institute of Biomolecular and Biomedical Research, University College Dublin, Dublin, Ireland; 2 Department of Clinical Pharmacology, Royal College of Surgeons in Ireland, Dublin, Ireland; 3 Department of Gastroenterology, Beaumont Hospital and Royal College of Surgeons in Ireland, Dublin, Ireland; 4 Department of Histopathology, Beaumont Hospital and Royal College of Surgeons in Ireland, Dublin, Ireland

## Abstract

**Background:**

Prostaglandin E_2 _(PGE_2_) is the major product of Cyclooxygenase-2 (COX-2) in colorectal cancer (CRC). We aimed to assess PGE_2 _cell surface receptors (EP 1–4) to examine the mechanisms by which PGE_2 _regulates tumour progression.

**Methods:**

Gene expression studies were performed by quantitative RT-PCR. Cell cycle was analysed by flow cytometry with cell proliferation quantified by BrdU incorporation measured by enzyme immunoassay. Immunohistochemistry was employed for expression studies on formalin fixed paraffin embedded tumour tissue.

**Results:**

EP4 was the most abundant subtype of PGE_2 _receptor in HT-29 and HCA7 cells (which show COX-2 dependent PGE_2 _generation) and was consistently the most abundant transcript in human colorectal tumours (n = 8) by qRT-PCR (ANOVA, p = 0.01). G0/G1 cell cycle arrest was observed in HT-29 cells treated with SC-236 5 μM (selective COX-2 inhibitor) for 24 hours (p = 0.02), an effect abrogated by co-incubation with PGE_2 _(1 μM). G0/G1 arrest was also seen with a specific EP4 receptor antagonist (EP4A, L-161982) (p = 0.01). Treatment of HT-29 cells with either SC-236 or EP4A caused reduction in intracellular cAMP (ANOVA, p = 0.01). Early induction in p21^WAF1/CIP1 ^expression (by qRT-PCR) was seen with EP4A treatment (mean fold increase 4.4, p = 0.04) while other genes remained unchanged. Similar induction in p21^WAF1/CIP1 ^was also seen with PD153025 (1 μM), an EGFR tyrosine kinase inhibitor, suggesting EGFR transactivation by EP4 as a potential mechanism. Additive inhibition of HCA7 proliferation was observed with the combination of SC-236 and neutralising antibody to amphiregulin (AR), a soluble EGFR ligand. Concordance in COX-2 and AR localisation in human colorectal tumours was noted.

**Conclusion:**

COX-2 regulates cell cycle transition via EP4 receptor and altered p21^WAF1/CIP1 ^expression. EGFR pathways appear important. Specific targeting of the EP4 receptor or downstream targets may offer a safer alternative to COX-2 inhibition in the chemoprevention of CRC.

## Background

Colorectal cancer (CRC) remains a leading cause of cancer death, with worldwide 1 million new cases each year and as many as half a million cancer deaths annually [1]. Cyclooxygenase-2 (COX-2) expression is increased in the majority of colorectal tumours [2] and this induction is associated with advanced tumour stage and correlates with poor clinical outcomes [3]. Non-steroidal anti-inflammatory drugs (NSAIDs), which inhibit COX activity, show anti-neoplastic effects *in-vitro *[4,5] and human studies have demonstrated their use to be associated with a reduced incidence of colorectal neoplasia [6,7]. While more recent studies have confirmed the chemo-preventive activity of COX-2 selective NSAIDs [8-10], it is also clear that long term therapy with COX-2 inhibitors is associated with an unacceptable increase in the risk of cardiovascular events [9,11].

The anti-neoplastic properties of NSAIDs result from the inhibition of prostaglandin generation, particularly prostaglandin E_2 _(PGE_2_), the most abundant *in-vivo *product of COX-2 activity in colorectal cancer cells [12,13]. The biological activity of PGE_2 _is mediated by binding to cell surface receptors. There are four subtypes of EP receptor (EP1, EP2, EP3, and EP4) with the majority localised to the plasma membrane. The binding of prostaglandins to cell surface receptors triggers changes in second messengers [14].

PGE_2 _modulates processes fundamental to tumour cell survival such as enhanced proliferation and resistance to apoptosis [4,15-17], however, the precise molecular mechanisms remain unclear. There is therefore a strong rationale to seek a more profound understanding of the downstream targets of COX-2 activity. Selective COX-2 inhibitors have shown promise as chemo-preventive agents [18], but their adverse cardiovascular effects have undermined their suitability for long term use [9,11]. Renewed attention must now therefore focus on the altered signalling occurring downstream of COX-2 in cancer as a source for new refined therapeutic targets.

## Methods

### Cell culture

HT-29 cells were purchased from the ATCC (Rockville, MD) and maintained in McCoy's 5 A medium containing 1.5 mM L-glutamine, 10% FBS, penicillin 100 U/ml and streptomycin 100 μg/ml. HCA7 cells were kindly donated by Susan Kirkland (ICRF, London, UK) and were cultured in DMEM with 10% FBS, supplemented with 1 mM sodium pyruvate and 100 μg/ml kanamycin. SC236, a selective COX-2 inhibitor was a gift from Dr. Peter Isakson (Searle, Skokie, IL). PGE_2 _was purchased from Cayman (St. Louis, MO). L-161982 (EP4A), a selective antagonist of the EP4 receptor was a kind gift of Merck Frosst, Canada [19]. PD153035 (EGFR tyrosine kinase inhibitor) was purchased from Calbiochem (La Jolla, CA). Wortmannin was purchased from Sigma Aldrich (Dublin, Ireland).

### Quantitative RT-PCR

Total RNA was isolated from cells and tissue following homogenisation in RNA lysis buffer (Qiagen Ltd. GmbH, Germany) supplemented with 1% β-mercaptoethanol. Extraction was performed using RNeasy™ Mini Kits (Qiagen Ltd. GmbH, Germany). Total RNA (1 μg) was reverse transcribed using Moloney Murine Leukaemia Virus (MMLV) reverse transcriptase (Promega, Madison, WI) according to the manufacturer's instructions. Gene expression was quantified by RT-PCR using SYBR Green Universal Master Mix (Roche Diagnostics Corp., Indianapolis, IN). Reactions were carried out in a 96 well format in the ABI 7700 Sequence Detector (Perkin Elmer/Applied Biosystems, UK). Results were then normalized to 18S rRNA amplified from the same cDNA mix. Sequences of the primer pairs used are listed below.

EP1- F ATG GTG GGC CAG CTT GTC

EP1- R GCC ACC AAC ACC AGC ATT G

EP2- F TGC CTT TCA CGA TTT TTG CA

EP2- R TTA ATT GAT AAA AAC CTA AGA GCT TGG A

EP3- F TCT CCG CTC CTG ATA ATG ATG TT

EP3- R TCT GCT TCT CCG TGT GTG TCT T

EP4-F CGA CCT TCT ACA CGC TGG TAT G

EP4-R CCG GGC TCA CCA ACA AAG T

Amphireg-F CTC GGG AGC CGA CTA TGA CTA

Amphireg-R GCT TAA CTA CCT GTT CAA CTC TGA CTG A

CyclinD1-F CTG GAG GTC TGC GAG GAA CA

CyclinD1-R TGC AGG CGG CTC TTT TTC

CDK4-F TGT TGT CCG GCT GAT GGA

CDK4-R AAA CAC AGG GTT ACC TTG ATC TC

CDK6-F CAA CTA GGA AAA ATC TTG GAC GTG AT

CDK6-R TTG GTT GGG CAG ATT TTG AAT

p21-F GCA GAC CAG CAT GAC AGA TTT CTA

p21-R GCG GAT TAG GGC TTC CTC TT

p27-F CCT GCA ACC GAC GAT TCT TC

p27-R TCT TAA TTC GAG CTG TTT ACG TTT GA

### Immunohistochemical staining for COX-2 and amphiregulin

Samples of formalin fixed, paraffin embedded tissue were deparaffinised and rehydrated in Xylene and Methanol. *Detection of COX-2*. Endogenous peroxidase activity was quenched with 0.3% H_2_O_2 _in Methanol. Specimens were blocked in 1.5% normal serum and then incubated with antibody to COX-2 (Rabbit polyclonal anti-human COX-2, Cayman Chemical Co.) diluted 1/200, followed by secondary antibody and ABC complex from Vectastain Elite kit (Vector Laboratories, Burlingame, CA). Sections were exposed to diaminobenzidine (DAB) (Sigma Aldrich, Dublin, Ireland) and counterstained with hematoxylin (Sigma Aldrich, Ireland) and mounted using DPX (BDH, Poole, UK). *Detection of Amphiregulin*. Antigen retrieval was performed by heating slides in 10 mM citrate buffer (pH6.0) for 4 minutes in a pressure cooker. Blocking was performed with goat serum for 30 minutes. Slides were then incubated with primary antibody to amphiregulin (rabbit polyclonal to Amphiregulin by Abcam, Cambridge, MA) diluted 1/200 in ChemMate™ antibody diluent (DakoCytomation, Galway, Ireland) for 1 hour and staining completed using the ChemMate™ DAKO Envision™ detection kit (DakoCytomation, Galway, Ireland) visualisation, counterstaining and mounting were performed as outlined above.

### Prostaglandin analysis and cAMP detection by enzyme immunoassay

Competitive enzyme immunoassay (EIA) was used to determine PGE_2 _levels in culture media in 96-well format by Assay Designs (Ann Arbor, MI, USA). Prostaglandin concentration was calculated using the optical density of the samples in relation to a standard curve generated by dilutions of a standard provided. Quantitative determination of cAMP concentration in cell lysates was performed using the cAMP (low pH) immunoassay by R and D Systems (Abingdon, Oxon, UK). Adherent cells were lysed in 0.1 N HCl for 10 min at 37°C and supernatants were assayed according to manufacturer's instructions in a 96 well plate. cAMP concentrations were calculated using a similar standard curve method.

### Cell proliferation (BrdU incorporation)

Cell proliferation assays were carried out using the BrdU (colorimetric) cell proliferation ELISA (Roche Applied Science, Dublin, Ireland) according to manufacturer's instructions. Cells were seeded at 5 × 10^3^/well in 96 well plates and following overnight serum starvation were treated with vehicle or drug. Amphiregulin neutralisation was with anti-human neutralising AR antibody (AR-ab) from Stratech Scientific (Soham, Cambridgeshire, UK).

### Cell cycle analysis

HT-29 cells were seeded at a density of 1 × 10^6^/well in 6 well plates and treated with drug or vehicle for 24 hours. Adherent cells were suspended using trypsin-EDTA for 3–5 min at 37°C and were fixed overnight in 75% ethanol at 4°C then washed and resuspended in a solution of PBS containing 0.1% Triton X-100, 0.05 mg/ml of DNase-free RNase and a 50 μg/ml propidium iodide (Molecular Probes, Leiden, NL) in the dark for 30 min. Cells were resuspended in PBS prior to analysis in a FACScalibur flow cytometer (Becton Dickinson, Oxford, UK) with measurement of fluorescence emission at >575 nm (FL3). Analysis of cell cycle distribution (DNA index) was performed using CellQuest™ (Becton Dickinson, Oxford, UK).

### Tumour collection

The protocol was approved by the Ethics (Medical Research) Committee of Beaumont Hospital, Dublin and all patients provided written, informed consent. Samples of colorectal tumour/normal were obtained from patients at the time of surgery and were immediately placed in RNAlater solution (Qiagen GmbH, Germany) or fixed in 10% formalin.

### Statistical analysis

A one-way analysis of variance (ANOVA) was used to examine overall differences between multiple groups, with Bonferroni multiple comparisons test. Two tailed paired students T-test was used to compare the means of paired samples. The statistical packages GraphPad InStat and GraphPad Prism were used. Statistical significance was set at a *P *value of less than 0.05, whereas a *P *value less than 0.005 was considered highly significant.

## Results

### EP receptor expression in HT-29 cells is similar to in-vivo expression in CRC

**Figure 1:**
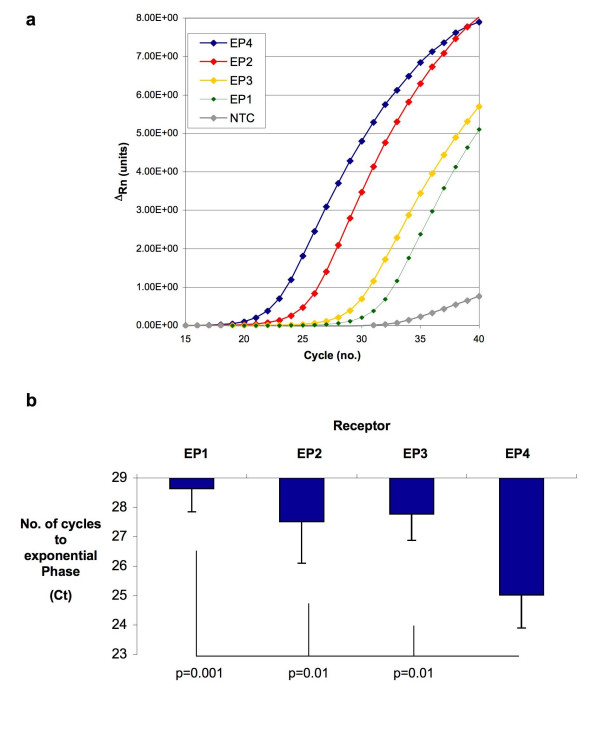
**EP4 Receptor Expression in HT-29 Cells and in Human CRC**. (*1a*). Representative amplification plot for the various EP receptors in HT-29 cells. The no template control (NTC) is indicated by the grey line. (*1b*) The number of cycles required to achieve exponential amplification of identical template from a panel of 8 colorectal tumours by qRT-PCR. The chart shows mean values +/- SEM for n = 8. Repeated measures ANOVA (overall) p = 0.0002, significant p values are shown and represent Bonferroni multiple comparisons test between selected groups.

EP receptor expression was assessed by qRT-PCR in the human colon cancer cell line HT-29 (n = 3) and EP4 receptor was the most abundant receptor subtype. A representative amplification plot is shown in Figure 1a with linear amplification of EP4 seen in identical template after the shortest number of cycles. The transcript for the EP2 receptor was less abundant than EP4 with significantly less transcript again for both the EP3 and EP1 receptors. A similar expression pattern of EP receptors was observed in HCA7 cells, which also generate PGE_2 _in a COX-2 dependent manner.

EP receptor expression was next assayed in human tumour samples (n = 8) with a similar expression pattern in all tumours studied. The EP4 receptor was consistently the most abundant receptor transcript (shortest number of cycles to exponential amplification in identical template, ANOVA p = 0.0002, Figure 1b). While the number of cycles to amplification was significantly less for the EP4 receptor than EP1 (p = 0.001) or EP2/EP3 (p = 0.01), no significant differences were noted in the abundance of the other EP receptors relative to each other. The relative abundance of the EP4 receptor in both cancer cell lines and in human tumour tissue suggested that signalling through this receptor might therefore be important in how PGE_2 _regulates tumour cell phenotypes.

### PGE_2 _generation in HT-29 is associated with cAMP generation via EP4 receptor

**Figure 2:**
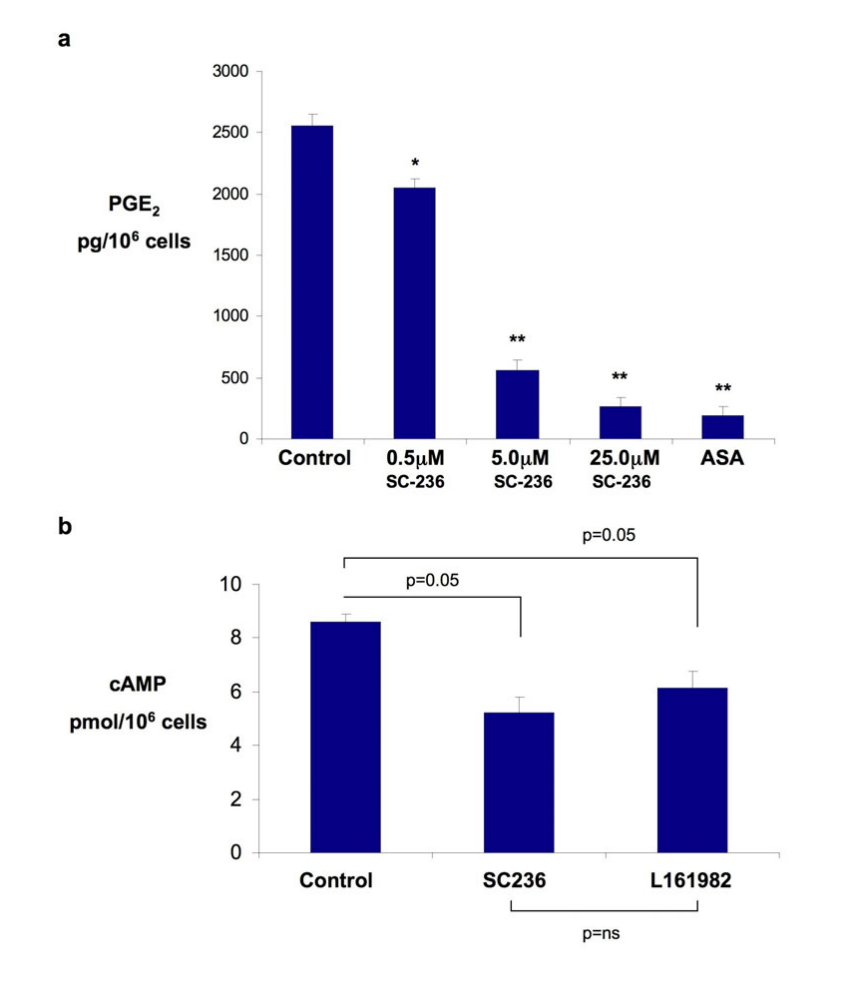
**COX-2 dependent generation of PGE_2 _and signalling through the EP4 receptor**. (*2a*) Generation of PGE_2 _in HT-29 cells over 15 minutes following treatment with various doses of the COX-2 inhibitor SC-236 or Aspirin (ASA, 200 μM) for 4 hours. Values are the mean +/- standard error of the mean (n = 3). One way ANOVA p = 0.0001; * p = 0.01 ** p = 0.001 (Bonferroni multiple comparisons test between selected groups). (*2b*) Intracellular cAMP concentrations following treatment with SC236 (5 μM) or L-161–982 (10 μM) for 30 mins. The chart shows mean values +/- SEM for n = 3. One way ANOVA (overall) p = 0.01, p values shown are for Bonferroni multiple comparisons test between selected groups.

While others have suggested that HT-29 cells lack the ability to generate prostaglandins through COX-2 activity [20], we confirmed that PGE_2 _is generated in a COX-2 dependent fashion in HT-29 cells and with EP4 receptor activation. Significant PGE_2 _generation by HT-29 cells was observed in control cells and this was reduced in a dose dependent manner by SC236, a COX-2 selective inhibitor (Figure 2a). The EP4 receptor signals by mediating changes in cAMP production via adenylate cyclase. The activity of this receptor (and thus indirectly EP4 protein expression) was demonstrated by measuring the generation of cAMP by cells with receptor modulation. Figure 2b shows the reduction in intracellular cAMP levels following both inhibition of PGE_2 _production by SC-236 and by a specific antagonist of the EP4 receptor (L-161982) confirming functional EP4 activity.

### PGE_2 _dependent regulation of cell cycle occurs through EP4 receptor

**Figure 3:**
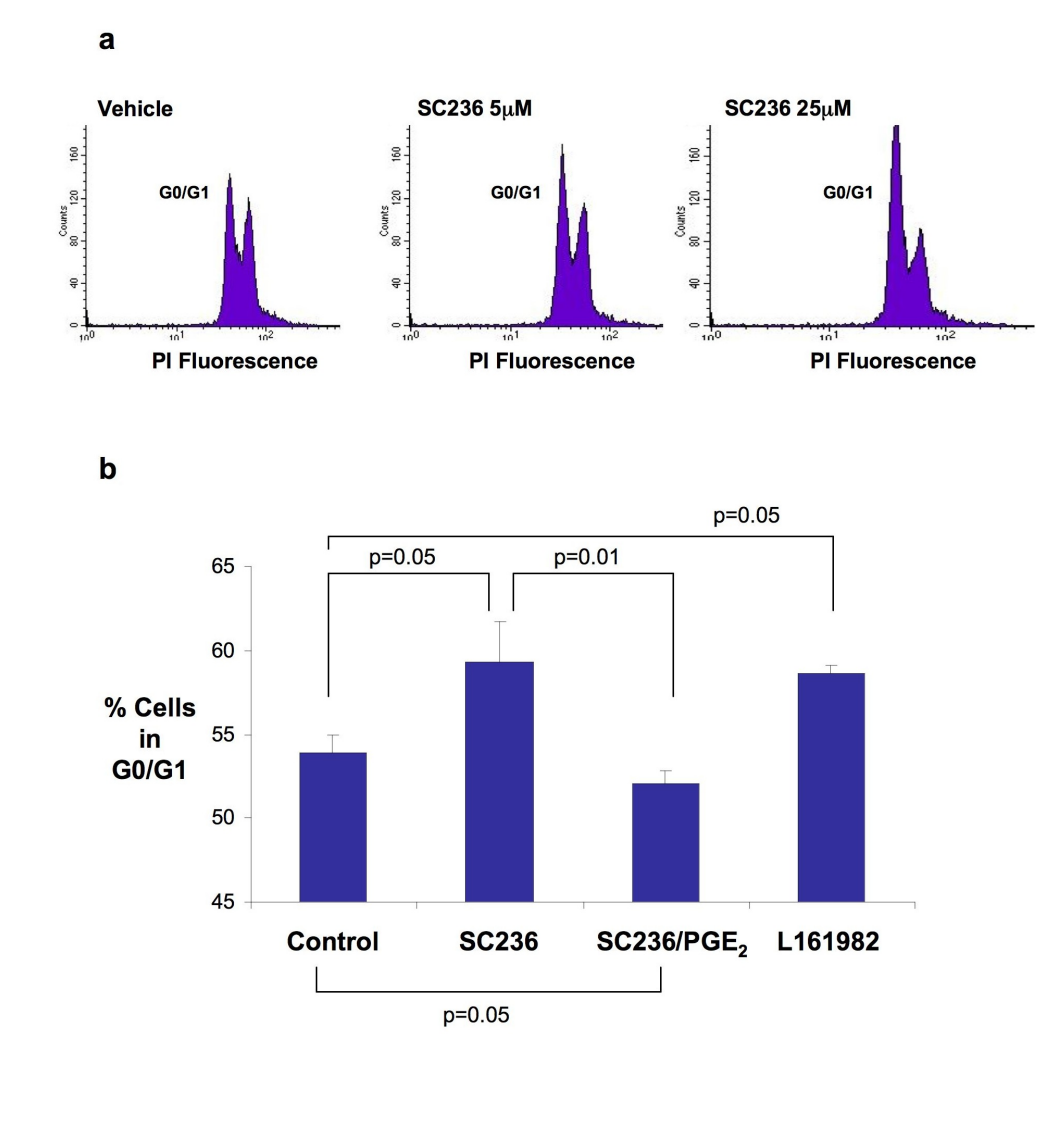
**Regulation of Cell Cycle Progression in HT-29 Cells**. (*3a*) Representative images of cell cycle distribution assessed by flow cytometry under the conditions outlined. (*3b*) Percentage of cells in the G1 peak in repeated flow experiments is shown (SC236 (5 μM) +/- PGE_2 _1 μM or L-161–982 (10 μM) The chart shows mean values +/- SEM for n = 5. One way ANOVA (overall) p = 0.01, p values shown are for Bonferroni multiple comparisons test between selected groups.

SC-236 increased the number of cells in the G_0_/G_1 _phase of the cell cycle over a range of doses. This G_0_/G_1 _arrest was much more marked with higher doses of the inhibitor (see Figure 3a). Interestingly, the effects of the inhibitor were not 'rescued' by co-incubation with exogenous prostaglandin at the higher dose. However, at doses of SC-236 in the low micromolar range (sufficient to abolish >90% of PGE_2 _production), a G_0_/G_1 _cell cycle arrest was observed which was reversed by co-incubation with exogenous PGE_2 _(Figure 3b). Cell cycle arrest was also seen on incubation with L-161982 suggesting that activation of the EP4 receptor by endogenous PGE_2 _plays a role in the regulation of cell cycle progression in HT-29 cells.

### Enhanced expression of p21^WAF1/CIP1 ^mediated by EP4 receptor

**Figure 4:**
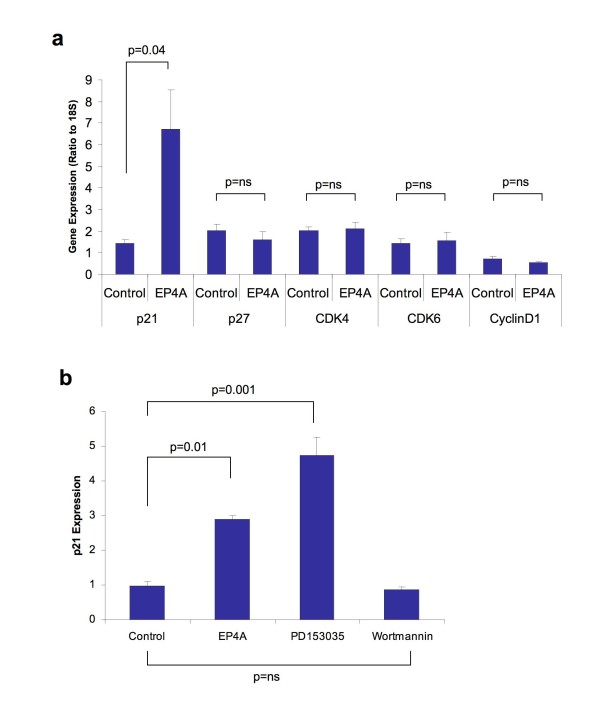
**Expression of p21^WAF1/CIP1 ^in HT-29 Cells is regulated by EGFR trans-activation through the EP4 receptor**. *(4a) *Expression of p21 by qRT-PCR in HT-29 cells following treatment with EP4A (L-161,982) 10 μM for 4 hours. Values shown are the Mean +/- SEM for n = 3. p values are for paired students t-test. *(4b) *Expression of p21 by qRT-PCR in HT-29 cells showing a comparison of the effects of EP4A (10 μM), a EGFR tyrosine kinase inhibitor (PD153025, 1 μM) and the PI3 Kinase inhibitor (Wortmannin, 1 μM). Values shown are the Mean +/- SEM (n = 3). One way ANOVA (overall) p = 0.0001, p values shown are for Bonferroni multiple comparisons test between selected groups.

The ability of selective EP4 receptor inhibition to modulate changes in the expression of cell cycle regulation genes was assessed to evaluate potential mechanisms for the observed phenotype. The expression of Cyclin D1, the cyclin dependent kinases CDK4 and CDK6 and finally the CDK inhibitors p21^WAF1/CIP1 ^and p27^KIP1 ^were evaluated. The results are summarised in Figure 4a. A significant induction in p21^WAF1/CIP1 ^expression was seen after 4 hours with EP4 receptor antagonism (mean change 4.4 fold, p = 0.04). Expression of the other cell cycle regulation genes remained unchanged. Similar induction in p21^WAF1/CIP1 ^expression was observed with PD153035, an EGFR tyrosine kinase inhibitor (p = 0.001), an effect not seen with the PI-3 kinase inhibitor wortmannin, suggesting transactivation of EGFR by EP4 as the likely mechanism for the induction of p21^WAF1/CIP1^.

### Relationship between EGFR and PGE2 in regulating of cell proliferation

EGFR transactivation appears important in PGE_2 _signalling and our results suggested a role in regulation of cell cycle progression genes and thus proliferation. PGE_2 _mediates EGFR activation by the release of EGFR ligands. Amphiregulin (AR) is known to be the most abundant EGFR ligand in HCA7 cells [21], which exhibit PGE_2 _dependent proliferation and therefore constitute an excellent *in-vitro *model to examining interplay between prostaglandins and EGFR.

**Figure 5:**
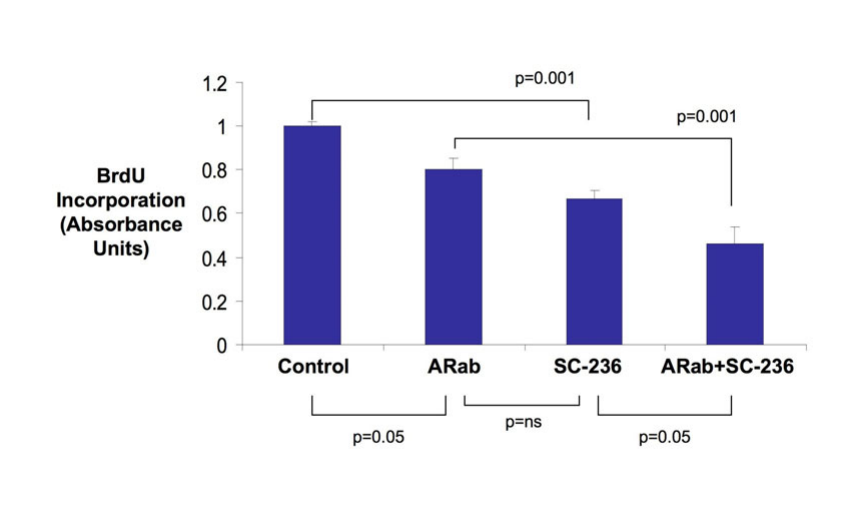
**Amphiregulin and COX-2 dependent cell growth in colon cancer cells**. Bar chart shows the effect on cell proliferation (as assessed by BrdU Incorportion) of a neutralising antibody to amphiregulin (ARab, 10 mg/ml), a COX-2 inhibitor (SC-236, 5 μM) or a combination of both for 24 hours. Values shown are the Mean +/- SEM for n = 5. One way ANOVA (overall) p = 0.0001, p values shown are for Bonferroni multiple comparisons test between selected groups.

The effect on HCA7 cell proliferation of a neutralising antibody to AR (ARab) both alone and in combination with SC-236 was evaluated. Effects of SC-236 on cell proliferation were again evident (33% mean reduction in proliferation, p = 0.001). At low concentration (0.1 – 1.0 mg/ml) the AR neutralizing antibody (ARab) had no effect (not shown), however at higher concentrations (10 mg/ml) a small effect (20% mean reduction, p = 0.05) on cell proliferation was noted (Figure 5). The effect of combined treatment with COX-2 inhibitor and ARab was greater than that of either ARab (p = 0.001) or SC-236 (p = 0.05) alone, resulting in a greater than 50% reduction in proliferation relative to control.

### Relationship between expression of COX-2 and amphiregulin in CRC

**Figure 6:**
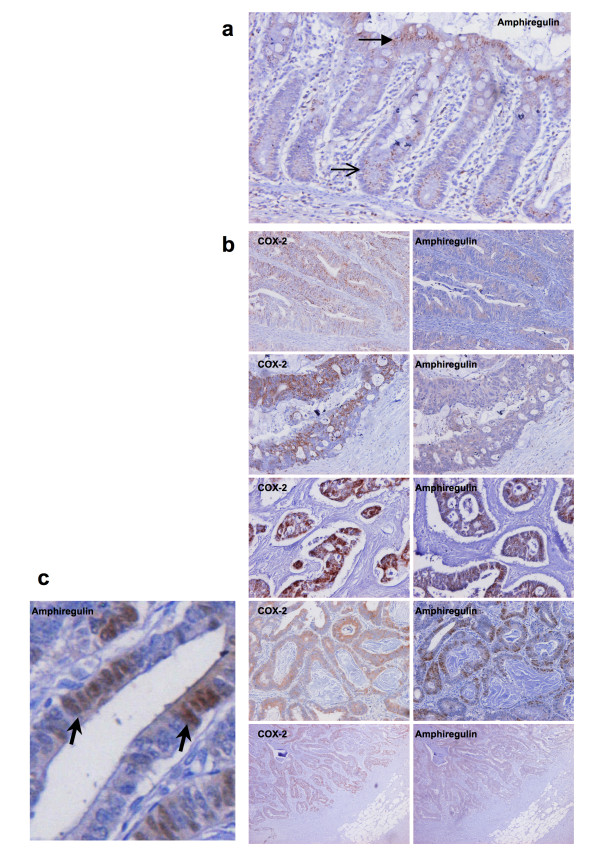
**Amphiregulin expression in normal colonic mucosa**. 6a. Positive staining (brown) for amphiregulin is seen in this section through a normal colonic mucosal gland. Staining is much stronger in the surface epithelial cells (solid arrow head) than in the crypt epithelial cells (open arrow head). **Relationship between amphiregulin and COX-2 expression in human colorectal cancer**. 6b. Representative paired images of immunocytochemistry for COX-2 (left) and amphiregulin (right) in a selection of tumour samples demonstrating a concordance in the localisation of positive staining within individual tumour specimens. **Nuclear localisation of amphiregulin expression in colorectal tumour**. Figure 6c (Bottom left panel) Focal areas of positive amphiregulin staining were observed in the nuclei of tumour cells (representative image with arrow head indicating positive nuclear staining).

The relationship between COX-2 and AR expression in human CRC was next examined. The expression of amphiregulin (AR) transcript was quantified in 10 tumour/normal pairs by qRT-PCR and correlated with expression of COX-2 in the same samples. AR expression was greater in tumour relative to normal in 7 of 10 patients (Mean fold difference = 4.2, p = 0.07 for paired t-test). A non-significant positive correlation was observed (Pearson r = 0.50, p = 0.13) between the tumour/normal differences for COX-2 and AR in the samples assayed. Closer inspection revealed higher than anticipated levels of AR expression in the normal colon (explaining the failure to observe a significant correlation). In an effort to better understand the relationship, immunohistochemistry on paired tumour and normal mucosa from an additional 20 patients was performed to evaluate expression of AR and COX-2. COX-2 immunoreactivity was virtually absent in normal colonic mucosa. AR expression was detectable in all of the normal samples, with a characteristic pattern of expression, being observed in the cytoplasm of the surface columnar epithelial cells while absent or reduced in crypt epithelial cells (Figure 6a). Differential expression of AR along the colonic crypt has been described previously [22]. COX-2 and AR expression in tumour tissue showed greater variability. Six of twenty cases (30%) showed absent or low level (<5% of cells positive) for both COX-2 and AR. In the remainder of patients, significant immunoreactivity for both molecules was observed and a concordance was observed in the localisation of positive staining within tumour specimens (representative images are presented in Figure 6b). COX-2 expression was observed exclusively in the cytoplasm of tumour epithelial cells. AR expression was mostly cytoplasmic, but focal areas of positive nuclear staining were also observed (Figure 6c).

## Discussion

COX-2 expression in colorectal tumours is biologically and clinically important [3,16] and PGE_2 _is the major product of COX-2 activity in cancer cells [12,13]. Four subtypes of membrane PGE_2 _receptor have been characterised (EP1–4), although the relative contribution of each of these to key signalling events in cancer has not been fully elucidated. We show that all of the EP receptor subtypes are expressed in human colorectal cancers and that EP4 receptor is predominant. We also demonstrate that the HT-29 cells share this relative distribution of receptors, validating its use as an *in-vitro *model.

Animal studies demonstrate that all four EP receptors are expressed in azoxymethane (AOM) induced tumours in rats [23]. Forced expression of COX-2 in murine mammary epithelial cells (with generation of PGE_2 _as the principal product) is associated with induction of EP 1, 2 and 4 receptors and down regulation of EP3 [24]. Knockout mice deficient in all four subtypes of EP receptor and with deletions of the DP, FP, IP or TP receptors have been generated. The formation of aberrant crypt foci (ACF) following AOM treatment was only different in animals with deletions of the EP1 [25] and the EP4 receptor [26]. While no difference in ACF formation with deletion of the EP2 receptor was detected; decreased polyp formation has been observed with deletion of the EP2 receptor in Apc^Δ716 ^mice [27].

There are limited data on the relative expression of these receptors in CRC in humans. A down-regulation of the EP3 receptor has recently been reported in human colorectal tumours [28]. Paradoxically, a study combining immunocytochemistry and in-situ hybridisation showed that EP3 and EP4 were the major receptors expressed (in association with COX-2 induction) in adenomatous polyps in patients with FAP [29]. A further study failed to demonstrate EP4 receptor or COX-2 mRNA induction in tumour specimens [30]. However, these observations are at variance with our findings and those of others [31]. EP4 receptor signalling modulates a tumourigenic phenotype in cancer cell lines and promotes metastatic potential *in-vivo *[31-33]. It has also also been demonstrated as important in the pro-neoplastic effects of PGE_2 _in a range of other human cancers; notably breast cancer where EP4 has been related to mediation of proliferation, invasion and metastasis [34,35]. Given its' relative abundance and functional activity, it seems reasonable to conclude that EP4 receptor mediates at least some of the important pro-neoplastic effects of PGE_2_.

Despite the ability of PGE_2 _to stimulate cancer cell growth [16], early data suggested that COX-2 over-expression in intestinal epithelial cells was associated with a paradoxical G_1 _delay [36]. Subsequent data suggest this occurs via prostaglandin independent mechanisms [37], perhaps representing an artefact of ectopic COX-2 expression. G_0_/G_1 _cell cycle arrest in cancer cells associated COX-2 inhibition has also been noted [38] and seems more plausible given the growth inhibitory effects of NSAIDs. We confirm the observation of G_0_/G_1 _arrest with COX-2 inhibitor treatment and demonstrate that the effect is PGE_2 _dependent. We observe that this effect is also produced by the EP4 receptor using a selective receptor antagonist (which shows similar effects on cellular cAMP concentration). Our observations are consistent with previous reports of modulation of cell growth in colon cancer cells through EP4 and of changes in susceptibility to apoptosis *via *EP4 receptor activation [16,39,40].

p21^WAF1/CIP1 ^is a cyclin dependent kinase inhibitor which indirectly regulates pRb phosphorylation and thus the G_1 _to S phase transition. Induction of p21^WAF1/CIP1 ^expression has been described in colon cancer cells following treatment with COX-2 selective inhibitors [38,41,42] and recent observations from other disease models suggest this is truly a prostanoid dependent event [43]. We demonstrate that selective induction of p21^WAF1/CIP1 ^expression is associated with EP4 receptor mediated cell cycle arrest. We also note repression of p21^WAF1/CIP1 ^expression in colorectal tumours (the majority of which express COX-2) samples in public expression datasets (Additional File 1). p21^WAF1/CIP1 ^is one of the few genes which shows consistent induction in expression in the rectal mucosa of patients treated with sulindac and deletion of p21^WAF1/CIP1 ^in a mouse model abolished the ability of sulindac to inhibit Apc-initiated tumourigenesis [44], observations which reinforce the hypothesis that p21^WAF1/CIP1 ^acts as a possible downstream effector of COX-2/PGE_2_/EP4 activity in CRC.

The EP4 receptor generates intracellular cyclic AMP (cAMP) via coupling to Gs proteins leading to activation of protein kinase A (PKA), phosphorylation of cAMP-response element binding protein (CREB) and PKA-dependent activation of extracellular signal-related kinase (ERK)[32]. However, in contrast to EP2 receptors (which also increase cAMP), EP4 receptors also activate phosphatidylinositol 3-kinase (PI3K) dependent signalling [45]. We did not observe p21^WAF1/CIP1 ^induction in HT-29 cells treated with the PI3K inhibitor wortmanin, however, p21^WAF1/CIP1^induction was seen with an EGFR tyrosine kinase inhibitor. PGE_2 _transactivates the epidermal growth factor receptor (EGFR) in HT-29 cells through c-Src-mediated release of the EGFR ligands [46]. This therefore seems the likely mechanism for PGE_2_/EP4 mediated changes in p21^WAF1/CIP1 ^expression.

To further clarify the role of EGFR transactivation, we focussed on the EGFR ligand amphiregulin (AR). AR expression in colon cancer cells (in culture) is increased by PGE_2 _via a cAMP/PKA dependent pathway [47], an effect therefore mediated through via EP2 or EP4 receptors. AR is the major EGFR ligand produced by HCA7 cells, where it acts as an autocrine growth factor [21]. L-161984 (EP4A) has also recently been shown to inhibit HCA7 proliferation [48]. We demonstrate the ability of COX-2 inhibition and AR neutralisation to inhibit HCA7 cell proliferation with an additive effect seen with a combination of both. This supports observations of the ability of PGE_2 _to synergistically enhance EGFR receptor tyrosine kinase signalling [49] and suggests a novel therapeutic approach. It has been recognised that EGF signalling may be important in sustaining elevated COX-2 expression[50], suggesting a positive feedback loop re-inforcing the increased activity of both pathways. Combined inhibition of COX-2 and EGFR may be a rational means to attempt to break this cycle and has shown promise in animal models [51,52]. Specific targeting of human EGFR with the monoclonal antibody cetuximab is already showing promise in trials in patients with metastatic CRC [53].

We also studied AR expression in human CRC and explored its relationship with COX-2 expression. In contrast to COX-2, AR showed significant expression in normal colonic mucosa. Interestingly, a pattern of differential expression along the colonic crypt is noted, similar to that previously described for the EP4 receptor [54]. Prior studies have shown increased AR expression in 50–70% of primary or metastatic colorectal tumours [55,56]. We observed a similar trend with 70% of our samples showing significant expression of both COX-2 and AR and concordance observed in the localisation of positive immunostaining within tumours. Interestingly, AR localisation was not confined to the cytoplasm of tumour epithelium as might be expected of a secreted glycoprotein (which acts as a ligand for EGFR). The significance of a nuclear localisation for AR has not been addressed although the presence of a nuclear localisation sequence in the AR protein has been noted and AR shows the ability to interact with nuclear proteins [57]. This raises the fascinating possibility that AR may act as a nuclear effector for PGE_2 _in cancer cells, a hypothesis which merits further evaluation.

## Conclusion

In conclusion, while selective COX-2 inhibition for chemo-prevention of CRC no longer appears a safe therapeutic option for the average risk patient due to the risk of vascular events [9,11], this strategy has shown clinical effectiveness in reducing incidence of colorectal neoplasia [8-10]. COX-2 remains an important target for CRC chemo-prevention, but future strategies must seek to target the activity of PGE_2 _in the colonic epithelium, while minimising effects elsewhere in the body[18]. Targeting of EP receptors, such as EP4 shows promise [58]. Other effectors such as amphiregulin and p21^WAF1/CIP1 ^also merit consideration to be targeted alone or in combination with other downstream molecules.

## Competing interests

The authors declare that they have no competing interests.

## Authors' contributions

GAD designed and performed cell culture experiments, RT-PCR, flow cytometry and immunohistochemistry, drafted and revised the manuscript. SMB helped design the study, performed cell culture experiments and reviewed the manuscript. ESM helped design the study, performed cell culture experiments and reviewed the manuscript. VM performed cell culture experiments with amphiregulin neutralisation and associated immunoassays. SCA assisted with experimental design and trouble shooting, analysis of results and reviewed the manuscript. EWK assisted with study design, optimisation and interpretation of immunohistochemistry and reviewed the manuscript. FEM assisted with experimental design, interpretation of data and review of the manuscript. DJF assisted with experimental design, interpretation of data, drafting and revision of the manuscript. All authors have read and approved the final manuscript.

## Pre-publication history

The pre-publication history for this paper can be accessed here:

http://www.biomedcentral.com/1471-2407/9/207/prepub

## Supplementary Material

Additional file 1 **Expression of p21^WAF1/CIP1 ^in colorectal cancer in public expression datasets**. Figure showing expression of p21^WAF1/CIP1 ^in colorectal tumour and normal colon in several public expression datasets. Click here for file
