# Supplementary figures and images for: Proneoplastic effects of PGE2 mediated by EP4 receptor in colorectal cancer
Source: BMC Cancer. 2009 Jun 26;9:207. doi: 10.1186/1471-2407-9-207 (PMC2714158; doi:10.1186/1471-2407-9-207)

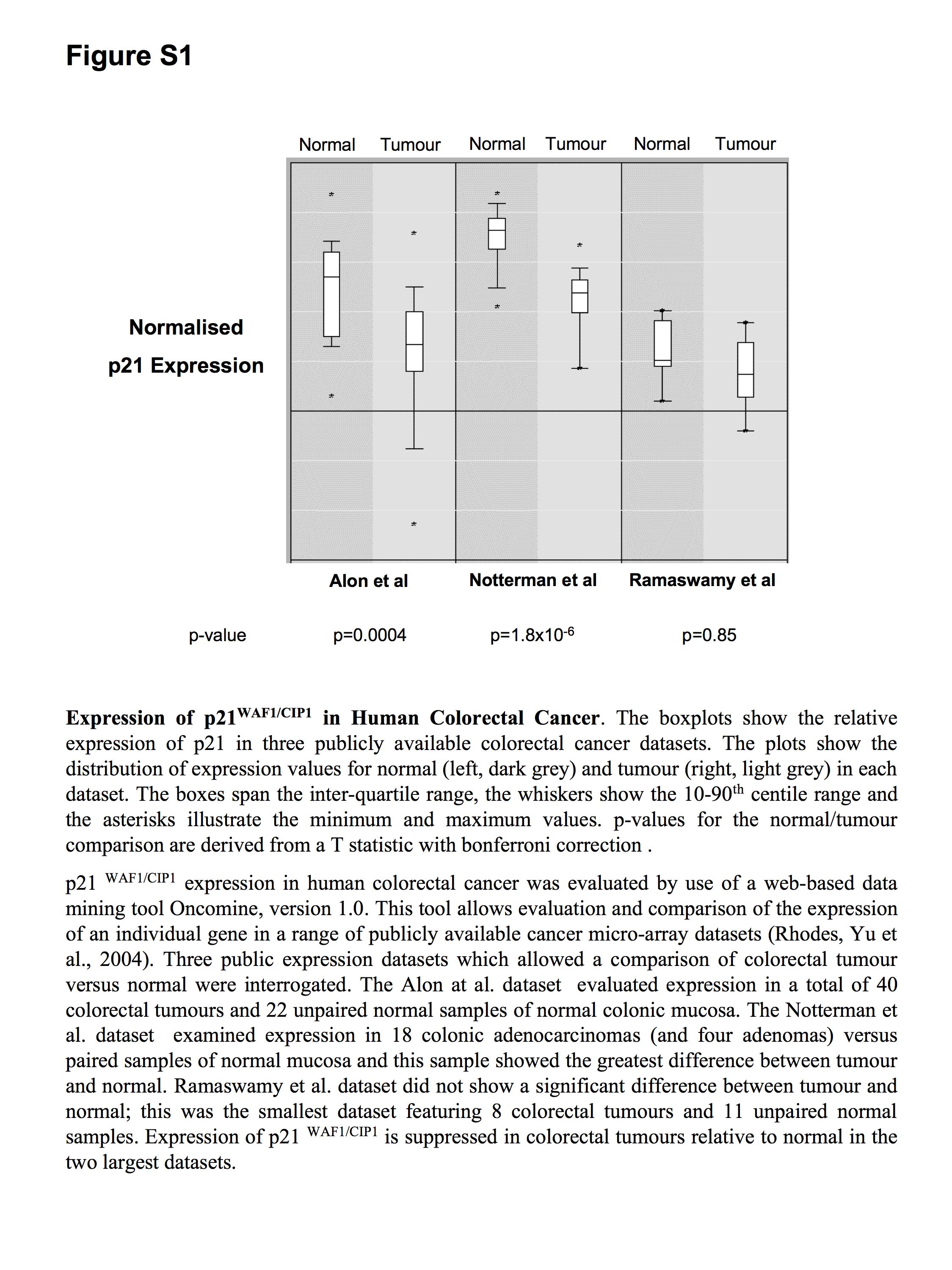

Supplement: Additional file 1 — Expression of p21WAF1/CIP1 in colorectal cancer in public expression datasets. Figure showing expression of p21WAF1/CIP1 in colorectal tumour and normal colon in several public expression datasets. [file 1471-2407-9-207-S1.jpeg]
